# Clinical analysis of germline copy number variation in DMD using a non-conjugate hierarchical Bayesian model

**DOI:** 10.1186/s12920-018-0404-4

**Published:** 2018-10-20

**Authors:** Velina Kozareva, Clayton Stroff, Maxwell Silver, Jonathan F. Freidin, Nigel F. Delaney

**Affiliations:** GenePeeks, Inc., 2067 Massachusetts Ave, Cambridge, MA, US

**Keywords:** Copy number variation (CNV), *DMD*, Carrier screening, Exome sequencing, Muscular dystrophy, Logit-normal distribution, Logistic normal distribution

## Abstract

**Background:**

Detection of copy number variants (CNVs) is an important aspect of clinical testing for several disorders, including Duchenne muscular dystrophy, and is often performed using multiplex ligation-dependent probe amplification (MLPA). However, since many genetic carrier screens depend instead on next-generation sequencing (NGS) for wider discovery of small variants, they often do not include CNV analysis. Moreover, most computational techniques developed to detect CNVs from exome sequencing data are not suitable for carrier screening, as they require matched normals, very large cohorts, or extensive gene panels.

**Methods:**

We present a computational software package, geneCNV (http://github.com/vkozareva/geneCNV), which can identify exon-level CNVs using exome sequencing data from only a few genes. The tool relies on a hierarchical parametric model trained on a small cohort of reference samples.

**Results:**

Using geneCNV, we accurately inferred heterozygous CNVs in the *DMD* gene across a cohort of 15 test subjects. These results were validated against MLPA, the current standard for clinical CNV analysis in *DMD*. We also benchmarked the tool’s performance against other computational techniques and found comparable or improved CNV detection in *DMD* using data from panels ranging from 4,000 genes to as few as 8 genes.

**Conclusions:**

geneCNV allows for the creation of cost-effective screening panels by allowing NGS sequencing approaches to generate results equivalent to bespoke genotyping assays like MLPA. By using a parametric model to detect CNVs, it also fulfills regulatory requirements to define a reference range for a genetic test. It is freely available and can be incorporated into any Illumina sequencing pipeline to create clinical assays for detection of exon duplications and deletions.

**Electronic supplementary material:**

The online version of this article (10.1186/s12920-018-0404-4) contains supplementary material, which is available to authorized users.

## Background

In recent years, analysis for copy number variants (CNVs), which have been demonstrated to be causal in a number of genetic disorders, has become a prominent component of clinical testing for diagnosis and prenatal screening [1–3]. However, while the vast majority of CNV analysis is performed using targeted microarray technologies [3, 4], many clinical tests rely predominantly on high-throughput sequencing in order to identify smaller causal variants more comprehensively [5].

In particular, carrier screening for recessive disease-associated variants is increasingly moving towards whole exome sequencing (WES) to detect single-nucleotide variants and small indels, forgoing broad CNV analysis [6–8]. This is concerning for several serious genetic disorders, such as Duchenne muscular dystrophy (DMD), where a large proportion of disease-causing mutations are copy number variants. In DMD (and the milder form Becker muscular dystrophy) approximately 75% of inherited causal mutations are copy number variants encompassing one or more exons in the *DMD* gene located on the X-chromosome [9, 10]. The majority of these variants do not encompass the entire gene, instead occurring in one of two known recombination hot spots, between exons 43 and 55, and exons 2 and 23.

To make WES more applicable for subsequent CNV analysis, several groups have worked on developing computational methods which can use targeted sequencing data to identify copy number variants [11–15]. However, although there have been some attempts to use these computational techniques in a clinical setting [5, 16, 17], a variety of limitations prevent most from being directly applicable to carrier screening.

Several of these methods focus on detecting larger CNVs in the context of tumor cell line studies, where factors like normal-cell contamination can affect identification and matched-normal samples are available [11, 12, 14]. Others rely on non-parametric models and are designed for large scale population studies [11–13]. Only a few have reported sensitivity and specificity levels for individual genes comparable to the levels obtained through microarray and other alternative methods. In contrast, genetic carrier screening involves germline mutation analysis without normal matches and typically provides only a small cohort of reference samples. Most of all, it requires a consistently high degree of sensitivity and specificity for both rare and common CNVs, even when only a small number of specific genes are being screened.

To address these shortcomings, we propose a parametric approach for detecting exon-level CNVs in a test sample, which uses a generative model for read depth data across targets in a small number of genes. We model read depth across these targets as multinomially distributed, allowing us to avoid having to explicitly correct for differences in capture efficiency and coverage biases caused by exon length or GC content across targets. To make the model more robust to the inherent variability in library preparation and sequencing, we incorporate a non-conjugate logistic-normal prior distribution into our model. We then implement a Markov Chain Monte Carlo (MCMC) approach in order to estimate posterior distributions for various copy number states across targets in the genes of interest. Like other techniques, our approach relies on read depth counts in a set of reference samples, specifically for estimation of the prior distribution parameters. These reference samples are assumed not to carry CNVs in the genes of interest and must be sequenced using the same pipeline as the samples to be tested.

We have implemented this model and the CNV detection pipeline in a python package called geneCNV. We then used the package to evaluate a set of samples with known CNVs in the *DMD* gene, and benchmarked our results against three other computational methods chosen to highlight a breadth of different approaches towards CNV detection (XHMM [13], CNVkit [14] and ExomeDepth [15]). In addition, all computational results were compared with results from multiplex ligation dependant probe amplification (MLPA), a standard clinical method for detection of CNVs in *DMD* [18, 19].

Currently, *DMD* is typically not included in many carrier screens, likely because of the additional processing required by CNV analysis [7]. However, with our pipeline and benchmarking analysis, we have demonstrated the ability to accurately detect CNVs in *DMD* in a clinical setting by using a parametric model and exome sequencing data.

## Methods

### A generative model for read depth data

In analyzing the proportion of read pairs mapping to each target of interest in *DMD*, we found significant correlation between samples processed using the same sequencing pipeline (Additional file 1: Figure S1). Based on this, we developed a generative model which treats target read pair counts as drawn from a multinomial distribution. Then to explicitly account for both the similarities and sample-to-sample variations across read pair count ratios, we incorporated a non-conjugate prior distribution for the multinomial probabilities. Though we considered a conjugate Dirichlet prior, we applied a multivariate logistic-normal distribution instead to account for any potential inter-target covariation and to more flexibly model variation in coverage across multiple samples.

Figure 1 describes the full model graphically, indicating the latent copy number states and latent target intensities which together define the overall target mapping probabilities. More explicitly, let *k* equal the number of targets of interest. Let *x*_*i*_ represent the unnormalized “intensity” for target *i*=1…*k* and assume the *x*_*i*_ for each sample are generated according to a multivariate logistic-normal process as follows [20]:

1. **v**={*v*_1_,…*v*_*k*−1_}∼*MVN*(**μ**,*Σ*)

**Fig. 1:**
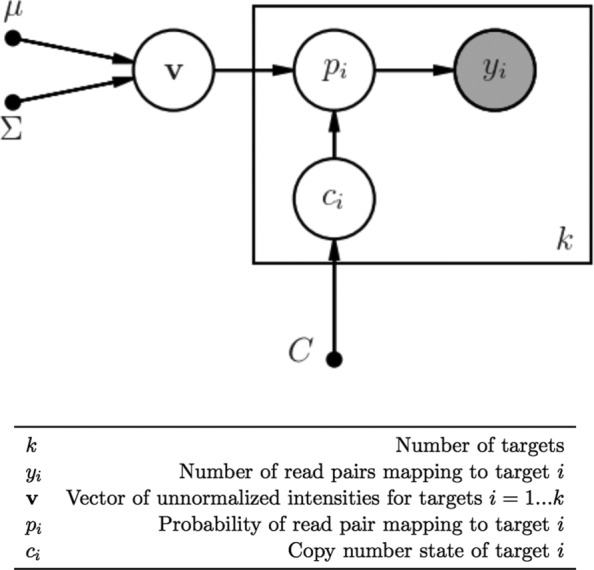
Generative model for read pair coverage during sequencing. Read pairs map to relevant targets during sequencing according to multinomial distribution with parameter **p**={*p*_1_,…,*p*_*k*_}. Note that **v** is drawn from multivariate normal with parameters *μ*,*Σ*, and *c*_*i*_ is drawn from multivariate discrete uniform distribution with support [ 0,*C*]
2. *v*_*k*_=0
3. $x_{i} = \frac {\exp {v_{i}}}{{\sum \nolimits }_{i=1}^{k} \exp {v_{i}}}$

Thus the prior distribution is fully specified by *μ* and *Σ*, which have dimension *k*−1 and *k*−1×*k*−1 respectively(for identifiability the last target intensity is kept constant). Defining the copy number state at each target as *c*_*i*_, we have the following for read pair counts *Y*={*y*_1_,…*y*_*k*_} for each sample:

$$Y \sim \text{Mult}(\mathbf{p}) \textrm{ where}\,\, p_{i} = \frac{c_{i}x_{i}}{\sum c_{i}x_{i}} $$

For the copy number states, we specify a discrete support representing the possible number of target copies (0,1,2,3). We found that expanding the support to include higher copy number states did not improve the performance of the model when doing germline analysis, though our implementation allows for an expanded support. To keep the model’s sensitivity high, we do not introduce a prior for the copy number states biased towards either 1 (for males) or 2 (for females), and instead use a discrete uniform prior. The unnormalized joint distribution corresponding to this model then becomes:

$$\Pr(\mathbf{C}, Y, \mathbf{v}; \mathbf{\mu}, \Sigma) \;\; \propto \;\; $$

$${} \exp{\left(-0.5(\mathbf{v} \,-\, \mathbf{\mu})' \!\Sigma^{-1} (\mathbf{v} \,-\, \mathbf{\mu})\right)} \left(\!\frac{1}{\sum c_{i} \exp{v_{i}}}\!\right)^{R} \!\prod_{i=1}^{k} \!(c_{i} \exp{v_{i}})^{y_{i}}$$

where $R = {\sum \nolimits }_{i} y_{i}$ represents the total number of read pairs in *Y*.

### Hyperparameter estimation

We implemented an expectation maximization algorithm first described by Hoff to fit the mean and covariance of the multivariate logistic-normal distribution based on read pair counts from 38 training samples [20, 21] (Additional file 1: Figure S1). In brief, the iterative process alternates between maximizing the conditional likelihood Pr(**v**|*Y*,*μ*,*Σ*) for each sample (to find the conditional mode of each **v**), and then maximizing the expectation of this likelihood with respect to *μ* and *Σ*. Thus the first step maximizes the following conditional likelihood:

1 $$ \frac{\exp\left({\sum\nolimits}_{i=1}^{k} v_{i} y_{i}\right)}{\left({\sum\nolimits}_{j=1}^{k} \exp v_{j}\right)^{R}} \exp \left(- \frac{1}{2} (\mathbf{v} - \mu_{a})^{T} \Sigma_{a}^{-1} (\mathbf{v} - \mu_{a}) \right)   $$

where *μ*_*a*_ and *Σ*_*a*_ are the values generated by the previous EM step. Then subsequent values (*μ*_*a*+1_,*Σ*_*a*+1_) are estimated through

$$\begin{array}{*{20}l} \arg \max_{\mu, \Sigma} \sum\limits_{i=1}^{m} \mathbb{E}[ \log \Pr(\mathbf{v}_{i}|\mu, \Sigma) |Y_{i}, \mu_{a}, \Sigma_{a}] \end{array} $$

where *m* is the number of training samples. This is approximated by minimizing

2 $$ \begin{aligned} m \log \vert \Sigma \vert\,+\, \sum\limits_{i=1}^{m} (\hat{\mu}_{i} \,-\, \mu)^{T} \Sigma^{-1} (\hat{\mu}_{i} \,-\, \mu) \,+\, \sum\limits_{i=1}^{m} \text{trace}\left(\Sigma^{-1} \hat{\Sigma_{i}}\right)  \end{aligned}  $$

This simplification takes advantage of the expectation of a quadratic form and the following multivariate normal approximation (3) to the conditional likelihood (1),

3 $$ \Pr(\mathbf{v}|Y, \mu, \Sigma) \approx MVN(\hat{\mu}, \hat{\Sigma})   $$

where $\hat {\mu }$ is the conditional mode of **v** and $\hat {\Sigma }$ is the negative inverse Hessian at the mode. Finally (2) is minimized by

$$\begin{array}{*{20}l} \mu_{a+1} &= \frac{1}{m} \sum\limits_{i=1}^{m} \hat{\mu}_{i} \quad \quad \text{and} \\ \Sigma_{a+1} &= \frac{1}{m} \sum\limits_{i=1}^{m} [(\hat{\mu}_{i} - \mu_{a+1})(\hat{\mu}_{i} - \mu_{a+1})^{T} + \hat{\Sigma}_{i} \end{array} $$

### Inferring copy number states

#### MCMC

Given the unnormalized joint distribution above and estimated hyperparameters, we can estimate the true joint distribution using a Markov Chain Monte Carlo technique. This also allows us to approximate the marginal posterior probability distributions for the copy number states. Examining the discrete copy number posterior probability distributions provides an intuitive measure of confidence (analogous to a high-density credible interval) that can be used as a decision criteria to make copy number variant calls.

Specifically, we implemented a variation of the Metropolis-within-Gibbs algorithm, where at each iteration, and for each target, we propose a new copy number state *c*_*i*_ drawn uniformly from its support and a new target intensity *v*_*i*_ conditioned on the most recent values for all other targets. To analyze convergence of the algorithm, we calculate and track the Gelman-Rubin potential scale reduction factor (PSRF) for the complete-data log likelihood and the *v*_*i*_ values, over steps of 5000 iterations and using a coarse optimization over burn-in proportion. As convergence criteria, we use the standard PSRF threshold of 1.1 for the log-likelihood and require at least 80% of *v*_*i*_ PSRFs to be less than 1.1 [22, 23]. After convergence, we calculate posterior probability distributions over the copy number states for each target from the iteration values.

#### Metastability error analysis

In addition to Gelman-Rubin convergence analysis, we account for some potential metastability error with an additional likelihood comparison step. Metastability error, when an MCMC simulation appears to have converged but has only reached a lower-likelihood metastable state, is caused by multimodality in the joint distribution space. In general, we reduce the chance of metastability error by running multiple chains and selecting overdispersed initial variable values (inherent in the first convergence analysis step). To further reduce the possibility of metastability error causing false positives, we compare the complete-data log-likelihood (*L*_*m*_) of the combination of most likely copy number states (comprised of the most likely copy number state in the posterior at each target) with the complete-data log-likelihood (*L*_*n*_) of the “normal” copy number state. For instance, in females, this would mean *c*_*i*_=2 for all targets. (Before comparison, the log-likelihoods are optimized with respect to target intensities, holding the copy number states constant at the values described above.) If *L*_*n*_ is significantly larger than *L*_*m*_, indicating metastability error, we repeat the MCMC simulation, until the difference *L*_*m*_−*L*_*n*_ surpasses a minimum (user-defined) threshold.

### Absolute copy number identification

Since our generative model cannot identify the absolute copy number state when all targets have equal copy number (as the relative frequency of all targets is equivalent), we incorporated “baseline” targets, which are assumed to be consistently representative of the normal genome-wide copy number. This model component relies on the concept that there are genes throughout the human genome which are highly dosage-dependent, and so are less likely to contain copy number variants in healthy individuals [24].

In a previous study using a similar sequencing pipeline [25], we identified several candidate baseline genes based on criteria including consistent average coverage across samples. For this study, we then selected a smaller set of baseline genes based on consistency of coverage relative to our targets of interest (in this case, *DMD* exons) across the samples used for training. Specifically, we ranked the original candidate genes by coefficient of variation *σ*_*i*_/*μ*_*i*_, where

$$\mu_{i} = \frac{1}{n}\sum\limits_{n} \frac{C_{\textrm{DMD}}}{C_{i}} \hspace{8pt} \text{for}~ n \textrm{ samples}$$

and *C*_DMD_ and *C*_*i*_ represent the total read pair coverage for *DMD* and gene *i* respectively. We selected seven genes with the lowest variation across subjects for a total of 112 additional “baseline” targets, which were included in the model and read pair counts as a single aggregated baseline. These genes and their corresponding regions and coefficients of variation are detailed in Additional file 2: Table S1. By including this aggregate baseline along with the targets of interest (thus increasing the dimensions of our hyperparameters and multinomial probability by one), we were able to accurately identify the absolute copy number states of the remaining targets. To accomplish this, during MCMC sampling, the copy number state of this aggregate baseline was kept constant and never updated. We also found that the final results were fairly robust to the exact number of genes selected for the aggregate baseline (Additional file 3: Table S2).

### Aggregation and final variant calling

Setting the posterior probability threshold for calling a copy number state not equal to the normal state helps determine the sensitivity and specificity of the test. For our study, we set a conservative threshold of 0.5 in order to maximize sensitivity, with a trade-off in specificity. This is equivalent to calling the copy number state with highest probability when the posterior distribution spans two states. Unlike other techniques, we did not attempt to aggregate targets before calling copy number state (through a hidden Markov model or other method), instead calling copy number state for each target individually and afterwards aggregating only those that matched in copy number. This choice was also motivated by our desire to increase sensitivity for small (single- or double-exon) CNVs.

### Sample selection and sequencing

For this study, a total of 43 volunteer saliva samples, along with 13 DNA samples obtained from the Coriell Institute (Coriell Institute for Medical Research, Camden, NJ) were used for model training and validation experiments. In order to benchmark our method’s ability to call DMD carrier status in females, the Coriell samples selected included all 9 samples of female carriers available from a genomic DNA reference panel created to allow for DMD genetic test development and quality control [26]. Saliva samples were collected, processed, and sequenced on the Illumina platform as described previously [25], with slight modifications. The sequencing of the saliva and Coriell research samples sequenced was performed on a NextSeq 500 sequencing system instead of a MiSeq, and in order to increase the genomic coverage of the *DMD* gene, samples were enriched with a custom mix-in panel containing a 2:1 ratio of baits from the Illumina TruSight One (TSO) panel (4813 genes) mixed with the Illumina Inherited Disease Panel capture bait set (a subset of 552 genes).

Of the volunteer samples, 38 samples were used in training the model, while the remaining five were used as test samples in validation experiments (Table 1). The selection of 38 samples to use for hyper-parameter estimation was based on an initial estimate of the sample size required. However, as described later simulations allowed us to demonstrate that only ∼35 samples would likely have been sufficient. All samples from Coriell were used as test samples in the validation experiments (Table 1, Additional file 4: Table S3). In addition, data from 13 volunteer samples previously sequenced using the TSO panel (according to the manufacturer’s protocol), was used in the training sample selection analysis (Additional file 1: Figure S1).

**Table 1:** Samples used for training and testing

Number of samples	DMD mutation	Sex	Source	Group
38	No known mutations	Female	Volunteer	Training
9	Various CNVs (Additional file 4: Table S3)	Female	Coriell	Test
4^a^	None	Female	Volunteer	Test
2^a^	None	Female	Coriell	Test
2^a^	Various CNVs (Additional file 4: Table S3)	Male	Coriell	Test
1^a^	No known mutations	Male	Volunteer	Test

^a^Used as negative control for software comparison only

^b^Used only for supplemental experiment (Additional file 10: Figure S7)

### Read pair coverage

Exon target coordinates were determined based on the intersection of TSO panel bait intervals and exon locations designated by Ensembl database transcripts for hg19 (for *DMD* transcript ENST00000357033.8, RefSeq NM_004006 was used). Coverage across exon targets was calculated using a module (included in the geneCNV package) to extract read pair counts from individual BAMs, where each counted molecule corresponds to a properly mapped pair of reads. Included reads were correctly oriented, with mapping quality ≥60 and insert length less than a designated merge distance (629 bp for *DMD*). Before computation, exons closer than the designated distance were merged to avoid repeated counting of read pairs that overlapped more than one exon. Reads flagged as PCR duplicates were excluded. In addition, due to insufficient and inconsistent coverage, exon 78 in *DMD* (chrX: 31144758-31144790) was excluded from all subsequent analysis. Summary coverage across the primary exons of *DMD* for training and test samples is visualized in Additional file 5: Figure S2.

### MLPA

Copy number states across *DMD* targets were confirmed for all samples analyzed in the software comparison through multiplex ligation-dependent probe amplification (MLPA). All amplification and processing steps were performed according to MLPA General Protocol and manufacturer protocol for the SALSA MLPA P034 DMD probe mix kit (MRC-Holland, Netherlands). Fragment separation and analysis was performed on the PCR products via capillary electrophoresis on the ABI 3130xl (Applied Biosystems, Foster City, USA). Data files were analyzed with Coffalyser.NET software maintained by MRC-Holland.

### Package Installation and Usage

geneCNV is a python package that provides a suite of programs to be run at the command line to train the model and test new samples for CNVs. Full documentation and tutorial for it is available online at http://genecnv.readthedocs.io/en/latest/index.html. Here, we very briefly review the main commands and workflows described by that documentation.

To begin using the package, one must first train the model hyperparameters. This requires a list of targets (e.g. exon locations) in the standard BED file format [27] which defines the genomic locations of regions whose copy number will be inferred by the program. Additionally, one needs a collection of BAM files from samples that are presumed normal for parameter training.

When selecting samples for model training, they should both capture the expected variation across samples, but also be drawn from similar enough samples that the parametric model described here is valid and there are no large categorical differences between them. For example, data could all come from a similar sequencing protocol (e.g. identical genomic extraction, bait set, instrument, etc.) but also capture the variation that is introduced as the protocol is repeated through time (e.g. different sequencing runs, lot numbers for reagents, technicians etc.). Additional file 1: Figure S1 shows correlations between samples used for training in this paper, as well as samples that were obtained using different baits for the exome enrichment. Data from the same bait set is well correlated, but data from different bait sets is strikingly different, indicating that data from one bait set should not be used to train a model that will analyze data from a different bait set.

Given a collection of BAM files containing sequence data from samples available for training and a corresponding BED file, the geneCNV package contains two commands that are used for hyper parameter training. The first, create-matrix will take a list of BAM files and genomic intervals and produce a file listing the number of read pairs mapping to each specified target. The second command, train-model will then analyze that matrix to infer the mean and variance hyperparameters for the model.

Once a model is trained, it can be used to evaluate new samples of unknown copy number state using the evaluate-sample command, which takes as input the hyperparameters output by the previous training command and coverage data from a new sample. As output, this command will produce a text file with the marginal posterior probabilities for the copy number state at each exon, another text file giving a summary of any CNVs detected, and a PDF graphic which provides a visualization of the copy number states as shown in Fig. 3c and d. Together, the three commands in the package provide a complete workflow from model training through analysis.

## Results

### Simulated parameter estimation error and classification performance

There are several potential sources of error in the model’s ability to accurately call CNVs, including poor estimation of the prior distribution’s hyperparameters, and subsequent inference error (of the copy number state probability distributions) introduced during MCMC sampling. As a proof of concept, we quantified the expected effects of varying read pair coverage and the number of training samples on the resulting error using simulated data.

**Fig. 2:**
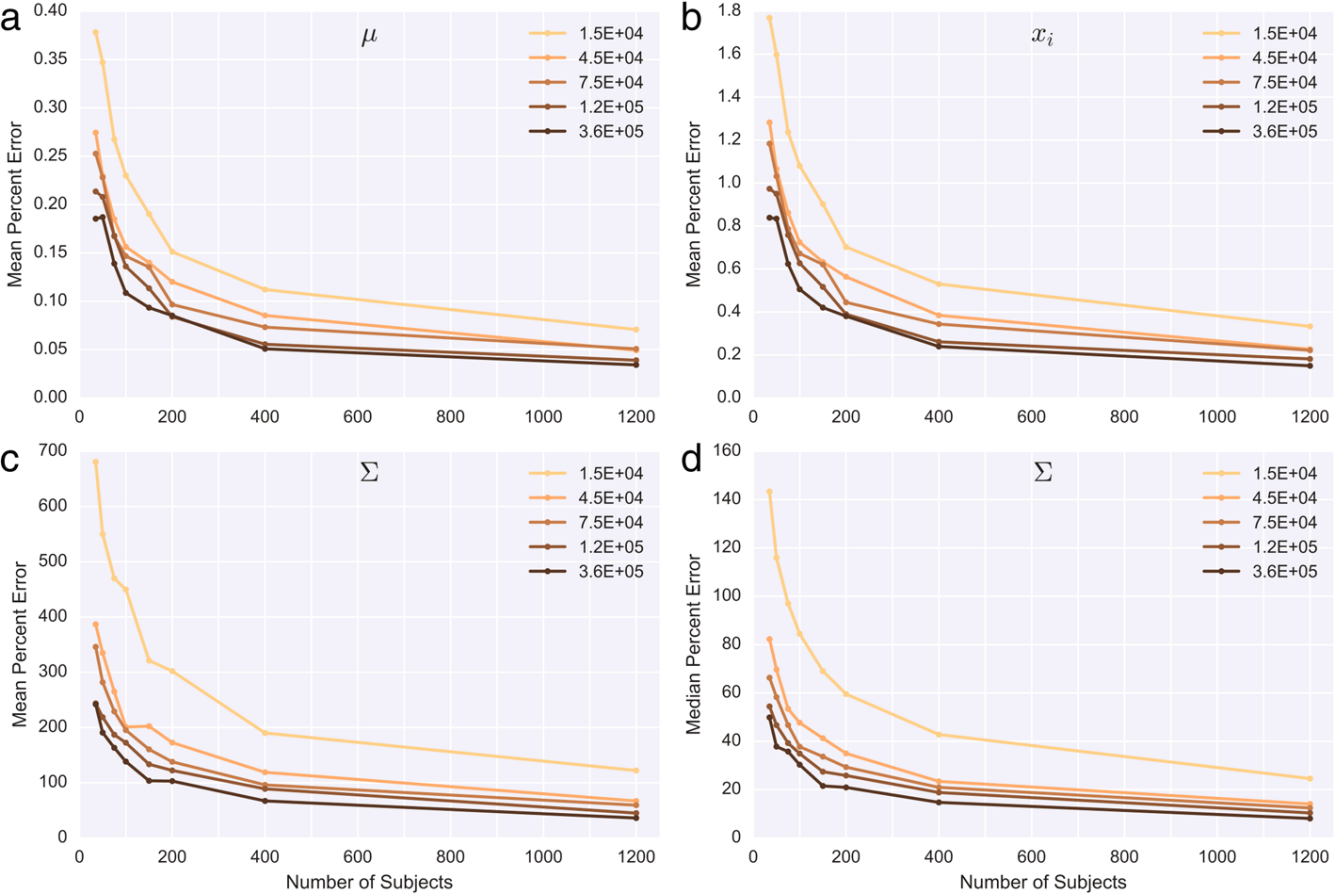
Error in multivariate normal hyperparameter estimation Original parameter (mean and covariance) values were derived from representative estimates for 79 targets across *DMD* (and an additional baseline target) using a cohort of high coverage samples. Each point represents the mean absolute percent error across 5 simulated sets of subjects at the coverage and cohort size indicated. For example, this is calculated as follows for *μ* and values of estimated $\hat {\mu }$: $100 * \frac {1}{5} {\sum \nolimits }_{k=1}^{5} \frac {1}{79}{\sum \nolimits }_{i} \vline \frac {\mu _{i} - \hat {\mu _{i}} }{ \mu _{i}} \vline $**a** shows percent error averaged across *μ*; **b** shows percent error averaged across the expected normalized *x*_*i*_ values; **c** mean and **d** median percent error across terms in *Σ*. Legend values indicate total read pair counts (including baseline targets) for each coverage level simulated

Figure 2 shows how the hyperparameter estimation error decreases as the both the number of samples and the total coverage per sample increases. We considered a single set of representative parameters, derived from mean and covariance values estimated from a cohort of high coverage samples. We then estimated these parameters using our EM training algorithm after simulating increasing numbers of read pairs for different numbers of samples. Error in estimation of the covariance terms decreased more significantly and consistently compared to error in the mean, though increasing coverage beyond 75,000 read pairs led to only a marginal continued decrease in error for both parameters. Similarly, increasing the size of the sample training set beyond 400 samples led to more modest decreases in estimation error of both the mean and covariance terms.

**Fig. 3:**
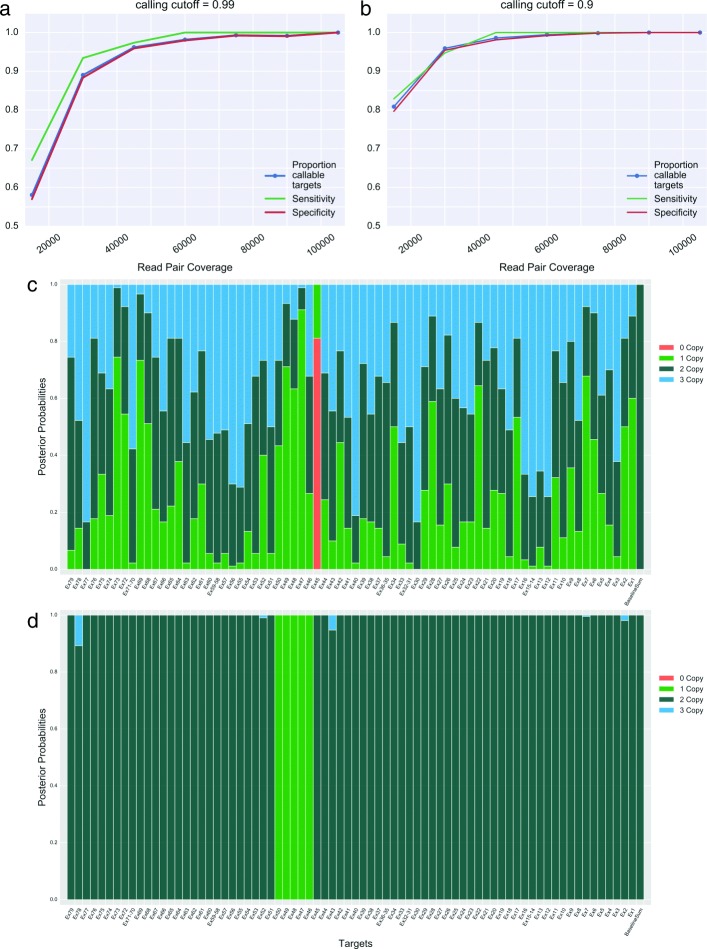
Classification performance with increasing read pair coverage Individual subject target intensities for nine simulated subjects were generated from hyperparameters estimated from a cohort of 38 volunteer subjects. True copy number states from nine Coriell test subjects (eight with CNVs and one negative control) were used to set multinomial probabilities before read pair coverage simulation. Panel x-axes indicate the total number of simulated read pairs mapping to the relevant exon targets (including the baseline targets). **a** and **b** indicate classification performance under the credible interval cutoffs of 0.99 and 0.9 respectively (i.e. targets where the highest-density interval of the chosen size overlaps two copy number states are not assigned a call). Callable targets are those assigned a final copy number call given the chosen cutoff. **c** and **d** display the copy number state visualization produced after MCMC sampling **c** indicates a typical result using a low read pair coverage (750 total read pairs). The underlying copy number states are unidentifiable. **d** shows results for a simulated sample with the same true copy number states as **c** but a total read pair coverage of 45,000 (approximately 20,700 at the targets of interest)

In terms of estimating the logistic-normal mean (and the resulting mean exon intensity values), even using just 35 training samples (and read pair coverage of 45,000) reduced the average percent error in the normalized *x*_*i*_ intensities to 1%. However, the percent error in the covariance terms was proportionally much higher, possibly because true covariation between targets (represented in the off-diagonal terms of the matrix) is likely very low on an absolute level. Analyzing the distribution of expected error in the covariance matrix revealed that there is a small number of terms with extremely high proportional error, and in fact, the median percent error is less than 60% for most cohort and coverage levels tested (Fig. 2) (Additional file 6: Figure S3 and Additional file 7: Figure S4). Thus, while limiting the mean percent error in the covariance terms to less than 100% would require an unrealistic cohort size and level of coverage for this number of targets, the majority of covariance terms could be estimated to within 80% of their true values with 35 training samples (and read pair coverage of 45,000).

Because the original parameters included a term representing the aggregate baseline, the total read pair count includes coverage outside of the main targets of interest (in this scenario, only about 46% of the total read pairs map to targets corresponding to exons in the gene of interest). Thus, coverage of 45,000 read pairs represents coverage at the level of approximately 21,000 for a gene similar to *DMD*. In terms of per-base coverage, this corresponds to an average read depth of about 250. Overall, the analysis indicates that at least 35 training samples with high coverage (> 200) across the gene of interest are needed to limit the parameter estimation error (particularly in the covariance terms) to a reasonable amount.

We also investigated the effect of increasing test sample coverage on the model’s ability to infer relative copy number states (Fig. 3). For this experiment, we assumed no estimation error in the prior parameters and generated all test sample target intensities from the same logistic-normal hyperparameters. We simulated nine different samples (eight with CNVs corresponding to those found in the Coriell test subjects, and one negative control) with levels of total read pair coverage varying from 15,000 to 105,000. In generating the copy number calls, we used credible interval cutoffs (instead of a threshold as described in Methods) to measure the proportion of targets we could call with reasonable certainty at each coverage level (callable targets). This analysis shows that even with a high calling cutoff, increasing test sample coverage to approximately 45,000 (∼21,000 for gene of interest) is sufficient to raise exon-level sensitivity and specificity above 95%, with marginal improvements as coverage increases beyond this level. At a slightly lower cutoff, all three metrics reach 100% at a coverage of 75,000 (∼34,000 for gene of interest). Thus, assuming the model has very low parameter estimation error, read pair coverage of 21,000 should generate accurate copy number calls.

In addition, Fig. 3 demonstrates the behavior of the MCMC results at very different coverage levels. At an extremely low coverage level (750 total read pairs), the resulting estimates for the copy number state distributions show a large amount of uncertainty, and the underlying true copy number states are unidentifiable. At a high level of coverage (45,000 read pairs total, with ∼20,700 mapping to the gene of interest), the copy number state distributions clearly indicate the simulated heterozygous deletion of five exons in this sample.

### Validation with samples heterozygous for CNVs in *DMD*

**Fig. 4:**
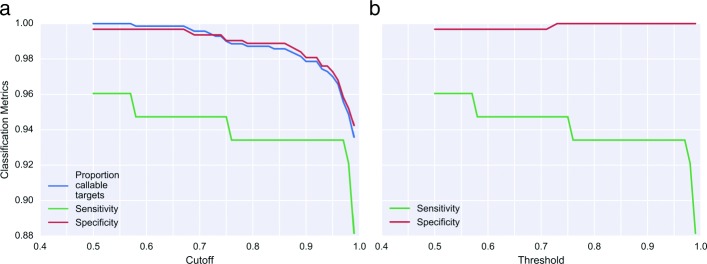
Sensitivity and specificity trade-off as cutoff and threshold vary exon-level classification performance of geneCNV model on nine Coriell samples, after hyperparameter training on 38 volunteer samples. Among the nine Coriell test samples here, there were a total of 77 affected exons and 634 unaffected exons, used in calculating sensitivity and specificity respectively. **a** shows the effects of varying the credible interval cutoff on the proportion of callable targets, true positives (sensitivity), and true negatives (specificity) for this test set. Exons where the highest-density interval of the chosen cutoff size spans two copy number states are given an “uncertain” call and not included in subsequent sensitivity and specificity analysis. **b** shows the effects of varying the threshold for abnormal copy number state probability (as defined in Methods) on sensitivity and specificity. Note that every exon is given a copy number call using this schema

To initially assess the model’s ability to accurately call CNVs in *DMD*, we used samples from nine Coriell subjects (eight of which are heterozygous for CNVs of various sizes, ranging from a single exon deletion to a 29 exon duplication). We estimated model hyperparameters from a set of 38 volunteer subjects sequenced using the same pipeline as the Coriell test subjects (Additional file 1: Figure S1). Figure 4 illustrates the model’s performance at different credible interval cutoff and threshold values. The proportion of certain calls at cutoffs of 0.9 and 0.99 were consistent with our simulation results, given the average *DMD* coverage (16,400) of these nine samples (36,000 across *DMD* and baseline targets). The observed sensitivity and specificity at these cutoff values were also roughly consistent with the simulation results in Fig. 3, indicating fairly low parameter estimation error from model training. As in the simulation, decreasing the cutoff consistently increased both sensitivity and specificity, though neither sensitivity nor specificity reached 1.0, even at the lowest possible cutoff. This indicated some noise in the final MCMC results (and potentially some error in the hyperparameter estimation), likely due to the lower coverage of these samples.

In calling complete copy number states, we used a conservative threshold of 0.5 instead of a cutoff (to generate calls across all targets), which achieved an exon-level sensitivity of 0.961 and a specificity of 0.997. Of the 77 exons included in the CNVs, 74 were correctly called by our model; the three false negatives were three non-contiguous exons in a 29-exon duplication (Table 2, Additional file 8: Table S5). At the subject level, where one only has to detect a change in any exon’s copy number to qualify the subject as a carrier, we observed perfect concordance between the geneCNV analysis and the known carrier statuses for these test samples.

**Table 2:** Inferred DMD copy number variants in test samples

		geneCNV exon-level calls				
Sample ID	Coriell/MLPA status	Targets	Class	Copy number	Mean posterior	Genomic Region
NA05117	Ex45DEL	Ex45	Deletion	1	1.0	X:31986445-31986641
NA04099	Ex49-52DEL	Ex49-52	Deletion	1	1.0	X:31747737-31854949
NA05159	Ex46-50DEL	Ex46-50	Deletion	1	1.0	X:31838081-31950354
NA07692	Ex1-18DEL	Ex1-18	Deletion	1	1.0	X:32536114-33229673
		Ex2-9	Duplication	3	0.9975	X:32715976-33038327
NA23087	Ex2-30DUP	Ex11-22	Duplication	3	1.0	X:32490270-32662440
		Ex24-25	Duplication	3	1.0	X:32481545-32482826
		Ex27-30	Duplication	3	1.0	X:32429858-32466765
NA23094	Ex35-43DEL	Ex35-43	Deletion	1	1.0	X:32305635-32383326
NA23099	Ex8-17DUP	Ex8-17	Duplication	3	0.9376	X:32563265-32717420
		Ex5^a^	Duplication	3	0.6521	X:3284140-328415141
NA04315	Ex44DEL	Ex44	Deletion	1	1.0	X:32235022-32235190
		Ex2^a^	Deletion	1	0.7831	X:33038245-33038327

Mean posterior represents the average posterior probability for the copy number status indicated across the targets called.

^a^Indicates an exon-level false positive result not consistent with MLPA CNV status. Mean posterior values for these exons are significantly lower than for other calls

### Comparison to other software methods

Although no single software technique has been established for standard clinical use in CNV analysis, we selected three other published techniques (representing diverse approaches to the problem of heterozygous CNV detection) for comparison with geneCNV. XHMM [13] uses a PCA-based approach to remove batch-level variation and a hidden Markov model to identify deletions and duplications. This approach makes the program well-suited for discovering relatively larger CNVs throughout whole exomes, using large cohorts of samples. CNVkit [14] utilizes a segmentation algorithm to identify regions differing in copy number, in addition to bias correction and reference normalization steps to subsequently call deletions and duplications. Finally, ExomeDepth [15] fits a beta-binomial model to the exon-level read-depth ratios of test and reference samples in order to identify copy number variants.

We used the same set of DMD-positive Coriell samples to measure exon- and subject-level sensitivity for each of the four methods, and included an additional six negative controls (two more Coriell samples and four volunteer samples) to better estimate subject-level specificity (Table 1 and Additional file 4: Table S3). Since the other algorithms are designed to utilize a larger number of capture targets, we expanded their interval inputs to targets throughout the entire X chromosome for our first comparison (Table 3). However, in calculating sensitivity and specificity, we only considered CNV calls in the *DMD* gene. Most prominently, we found that the other three tools did not generate any false positive calls, so their specificities on an exon-level were higher than that of geneCNV, although on the subject-level, all of the methods had a specificity of 1.0. However, XHMM and CNVkit did not detect several CNVs in different subjects, resulting in significantly lower subject-level sensitivities. Both geneCNV and exomeDepth identified the CNVs in all positive samples, though exomeDepth did outperform geneCNV slightly in terms of exon-level sensitivity.

**Table 3:** Software results comparison

	geneCNV	exomeDepth	XHMM	CNVkit
Subject-level sensitivity	1.0	1.0	0.75	0.5
Exon-level sensitivity	0.961	0.974	0.922	0.857
Subject-level specificity	1.0	1.0	1.0	1.0
Exon-level specificity	0.998	1.0	1.0	1.0

The two CNVs not detected by either XHMM or CNVkit were both single-exon deletions, which suggests fundamental limitations in these techniques’ power to call small heterozygous CNVs. As CNVkit relies on a segmentation algorithm, it is likely that very small CNVs would be filtered out as noise instead of separate segments, and the authors in fact reported poorer sensitivity on small CNVs. Similarly, XHMM’s combination of read-depth normalization and hidden Markov model for identification of contiguous CNVs could result in small CNVs being discounted as noisy data. Manually increasing the Markov model’s exome-wide CNV rate did not improve XHMM’s sensitivity, although the relatively small cohort used in this study would also limit its performance.

Like geneCNV, exomeDepth identified CNVs (with greater than 80% overlap) in all eight positive samples. The two approaches are similar in that, fundamentally, they attempt to detect deviation from an expected read count at a particular exon; in geneCNV, this expected read pair count is calculated relative to other targets in the test sample, while in exomeDepth, the expected read count is calculated relative to the same target in an aggregated set of reference samples. This model distinction helps illustrate a key difference in how performance can change for the two methods as the number of targets increases. Adding additional targets during geneCNV’s parameter estimation step increases the number of covariance terms to estimate, and thus the potential estimation error (Additional file 9: Figure S6), whereas additional targets in exomeDepth provide more data observations on which to fit the beta-binomial model.

We then performed a second comparison where we limited the target inputs to all software techniques, simulating a limited gene panel (Table 4). We included data for all exons in *DMD* and the seven genes used as baselines during absolute copy number identification (Methods). In this comparison, geneCNV had the highest sensitivity on both the exon and subject levels. Sensitivity decreased overall for the other three methods, though their levels of specificity did not change. In particular, exomeDepth had significantly lower exon- and subject-level sensitivities, as it did not identify a 29-exon duplication in one of the samples. These results indicate greater advantage (in terms of achieving both high sensitivity and specificity) in using geneCNV with smaller targeted gene panels, where the total number of genes is limited.

**Table 4:** Software results comparison (limited gene panel)

	geneCNV	exomeDepth	XHMM	CNVkit
Subject-level sensitivity	1.0	0.875	0.75	0.5
Exon-level sensitivity	0.961	0.623	0.883	0.857
Subject-level specificity	1.0	1.0	1.0	1.0
Exon-level specificity	0.998	1.0	1.0	1.0

## Discussion

In this study, we developed and validated a novel computational method for identifying copy number variants from targeted exome sequencing data using a generative Bayesian model. Unlike most other methods, our generative model is intended to be representative of the underlying reactions, including paired-end read alignment, during a typical hybrid-capture sequencing pipeline. An advantage of our approach is that the hyperparameters can be used to define an expected (or reference) range of normal samples, which must be estimated for tests in CLIA certified labs [28]. Additionally, although in many contexts this reference range is a “nuisance parameter” which unlike the copy number state is not directly of interest, defining the expected range of normal variation across replicate samples with these hyperparameters provides a direct way to perform quality control management and detect deviations in clinical sequencing processes.

Since our technique models target alignment with a multinomial distribution, an important consideration was the prior distribution for the multinomial parameters. Our simulation results indicate that using a multivariate logistic-normal distribution yields accurate copy number identification when the prior parameters are well-estimated and coverage is sufficiently high (approximately 21,000 read pairs across targets of interest, or an average of 275 read pairs per exon). The accuracy of the prior parameter estimation is sensitive to the number of samples in the reference set, in addition to these samples’ coverage levels. Assuming a similarly high level of coverage, the prior mean can be accurately estimated with as few as 30 reference samples. The prior covariance can be reasonably estimated with 30-50 samples, although additional reference samples (and increased coverage) will improve parameter estimation.

We then demonstrated the method’s utility as part of a downstream clinical analysis of copy number variation in the context of carrier screening for the *DMD* gene. We used geneCNV to detect CNVs in nine Coriell samples with known carrier statuses (including eight with large deletions or duplications and one negative control). On a subject level, we found complete concordance between the overall carrier statuses of these samples (which were independently confirmed by MLPA), and the mutation calls generated by our program. Across the total number of affected and unaffected exons in these nine samples, we observed an overall sensitivity of 0.96 and a specificity of 0.998, indicating almost complete agreement between geneCNV’s mutation calls and actual copy number state on an exon level as well.

Compared to existing software designed to detect CNVs using exome sequencing data, geneCNV tends to be more sensitive to small deletions and duplications. This is consistent with the idea that many published methods do not focus explicitly on CNV detection as part of clinical germline analysis, and are instead better suited for goals such as tumor analysis and rare CNV discovery. However, the package exomeDepth achieved results very similar to ours and was the only compared technique with higher performance on both exon-level sensitivity and specificity.

ExomeDepth’s approach is also most related to ours from a modeling perspective, but an important difference is that increasing the number of targets improves the fit of exomeDepth’s model. Conversely, limiting the number of targets tested simultaneously with geneCNV increases parameter estimation accuracy. Indeed, when using input data from only a few genes, geneCNV achieved higher sensitivity for CNVs in *DMD* than exomeDepth, which suggests potentially distinctive use-cases for geneCNV and exomeDepth. For whole exome sequencing data, or very large sequencing panels, exomeDepth is likely to have comparable or better performance than geneCNV in identifying CNVs across large numbers of targets (>100). With fewer targets and limited gene panels though, geneCNV is more likely to achieve results closest in sensitivity and specificity to clinically used assays.

Using geneCNV for clinical CNV analysis in *DMD* demonstrates another advantage of the model, which allows for testing of targets on the sex chromosomes in addition to autosomal targets. As long as baseline normalization is included, and the model is trained on female samples, absolute copy numbers can be estimated for targets across all chromosomes for both male and female test samples (Additional file 10: Figure S7), allowing the model to both detect female carriers of *DMD* mutations and diagnose affected males.

Finally, it is important to note that geneCNV’s model is applicable for CNV detection for diseases and genes outside of *DMD*, provided that users train the model on an appropriate training set. As for DMD, any samples used in these training sets should not contain CNVs in the targets of interest, and should be processed using the same sequencing pipeline in order to provide accurate results for test samples. In addition, any baseline targets should be reevaluated for consistency of coverage relative to the disease-relevant targets.

## Conclusions

This validation of our computational technique for CNV detection helps expand the potential utility of whole exome and targeted panel sequencing used in carrier screening. This is particularly true for genes like *DMD* which have thus far been inadequately covered by most existing carrier screens. By incorporating our technique into an existing high-throughput sequencing pipeline, clinicians can more easily conduct accurate CNV analysis for multiple disease-causing genes without relying on additional laboratory assays. Notably, because geneCNV uses an explicit parametric model, its hyperparameters can be used to define the reference range required for CLIA approved laboratory tests [28], allowing the technique to be readily applied in these laboratories.

## Additional files

Additional file 1 **Figure S1.** Pairwise sample correlation for normalized *DMD* target coverage. (PDF 213 kb)

Additional file 2 **Table S1.** Selected baseline genes and coefficients of variation. (PDF 98 kb)

Additional file 3 **Table S2.** Effects of baseline selection on exon-level sensitivity and specificity. (PDF 46 kb)

Additional file 4 **Table S3.** Coriell samples used for validation and supplemental experiments. (PDF 53 kb)

Additional file 5 **Figure S2.** Read pair coverage for training and test samples. (PDF 483 kb)

Additional file 6 **Figure S3.** Covariance estimation error. (PDF 202 kb)

Additional file 7 **Figure S4.** Covariance estimation error distributions. (PDF 169 kb)

Additional file 8 **Figure S5.** Results and coverage comparison for subject NA02387. (PDF 291 kb)

Additional file 9 **Figure S6.** Estimation error with target number. (PDF 204 kb)

Additional file 10 **Figure S7.** CNV identification in male subjects. (PDF 253 kb)

## Abbreviations

CLIA: Clinical laboratory improvement amendments
CNV: Copy number variant
DMD: Duchenne muscular dystrophy
EM: Expectation-Maximization
MCMC: Markov chain monte carlo
MLPA: Multiplex ligation-dependent probe amplification
MVN: Multivariate normal distribution
NGS: Next generation sequencing
TSO: TruSight one
WES: Whole exome sequencing
