# Supplementary material for: Clinical analysis of germline copy number variation in DMD using a non-conjugate hierarchical Bayesian model
Source: BMC Med Genomics. 2018 Oct 20;11:91. doi: 10.1186/s12920-018-0404-4 (PMC6195989; doi:10.1186/s12920-018-0404-4)
Supplement: Supplementary file 1 — Figure S1. Pairwise sample correlation for normalized DMD target coverage. (PDF 213 kb) [file 12920_2018_404_MOESM1_ESM.pdf]

1 Supplementary method: Selection of training samples

In order to train the model and estimate hyperparameter values, geneCNV requires a set of presumed normal samples sequenced using the same pipeline and capture technology. For our validation experiments, we identified 38 volunteer samples that showed similar target coverage (and were sequenced with the same bait set) in training the model. We examined pairwise sample correlations for normalized coverage across *DMD* targets in these training samples, in addition to the eight CNV positive validation samples, and 13 samples sequenced with a different bait set.

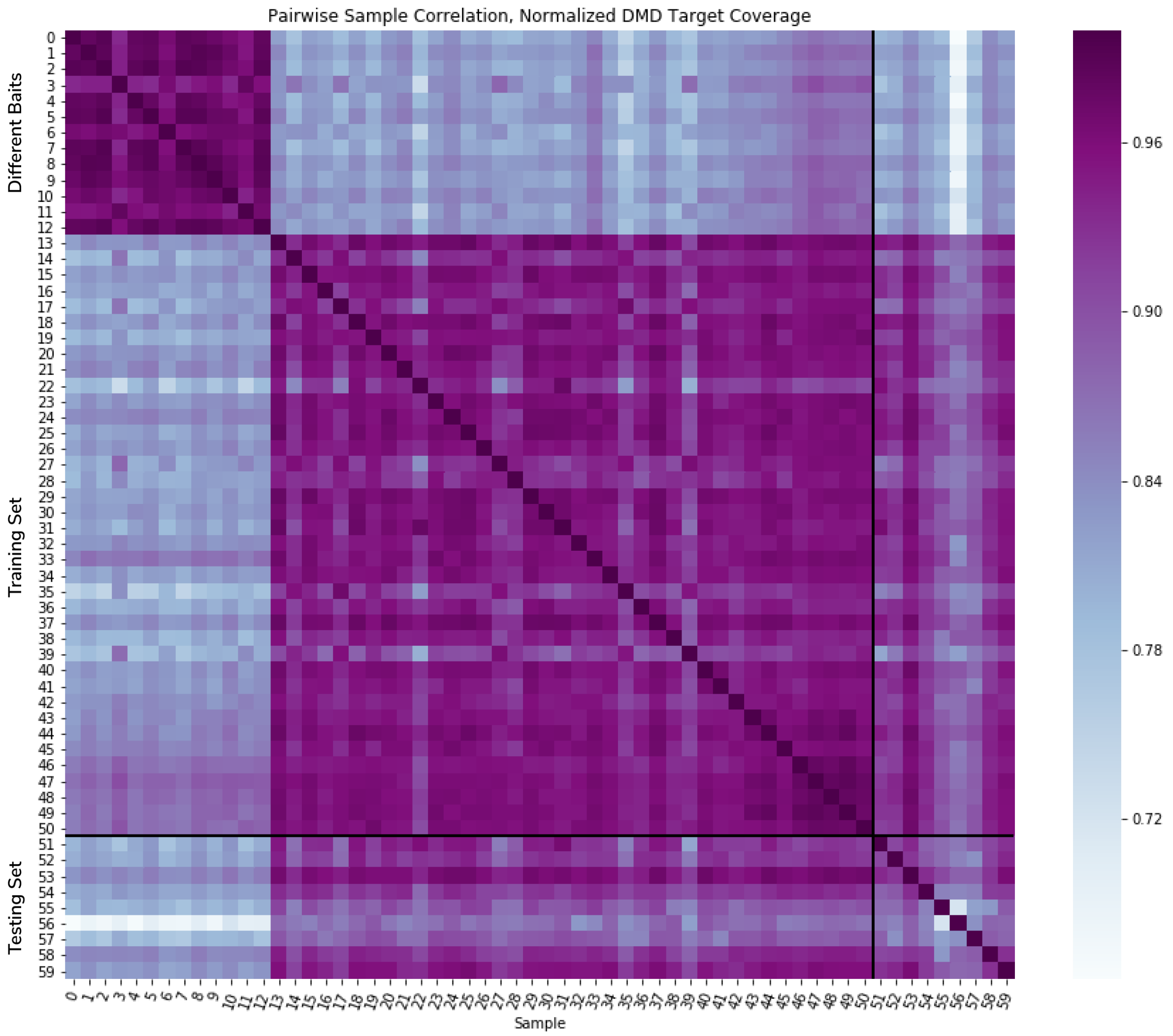

Figure S1: Pairwise sample correlation for normalized *DMD* target coverage Coverage across *DMD* exons was computed for 60 samples sequenced with two distinct capture sets (one as described in Methods, one with an older version of the TSO panel). Individual target coverage was then normalized by total gene coverage and sample-to-sample correlations were calculated pairwise.

Figure S1 displays these correlations, demonstrating a relatively high degree of correlation among the training and testing samples, compared to the samples sequenced with a separate bait set. As expected, there is some observable variation even among samples using the same bait set, likely due to different batch-level effects. To estimate model parameters as generally as possible, we did not exclude any of these samples, though outliers with any pairwise correlations < 0.8 could be excluded from a training set. In addition, it should be noted that test samples with larger CNVs (such as sample 56, which contains a 29 exon duplication) will show

lower levels of overall correlation with other samples.
