# Supplementary material for: Clinical analysis of germline copy number variation in DMD using a non-conjugate hierarchical Bayesian model
Source: BMC Med Genomics. 2018 Oct 20;11:91. doi: 10.1186/s12920-018-0404-4 (PMC6195989; doi:10.1186/s12920-018-0404-4)
Supplement: Supplementary file 2 — Table S1. Selected baseline genes and coefficients of variation. (PDF 98 kb) [file 12920_2018_404_MOESM2_ESM.pdf]

Table S1: Selected baseline genes and coefficients of variation

Gene	Mean Coverage (DMD-relative)	Coefficient of Variation (σ/μ)	Region
FH	9.521	0.037	1:241661107-241683042
GFM1	5.358	0.041	3:158362403-158409276
FASTKD2	7.239	0.045	2:207631397-207656546
ACADM	8.042	0.045	1:76190464-76228468
AGL	2.997	0.050	1:100316578-100387227
SLC35D1	9.892	0.050	1:67470002-67519716
ALG6	6.794	0.052	1:63836628-63902711
Aggregate	0.880	0.040	

Mean and coefficient of variation for aggregate baseline are calculated based on total coverage across all baseline genes, such that $\mu_A = \frac{1}{38} \sum \frac{C_{\text{DMD}}}{\sum_i C_i}$. Note that the total number of baseline genes selected was limited so as to keep μ_A relatively close to 1, while holding σ_A/μ_A low. Specific genomic regions for each of the 112 targets comprising these genes can be found at `http:/github.com/vkozareva/geneCNV/`.
