# Supplementary material for: Clinical analysis of germline copy number variation in DMD using a non-conjugate hierarchical Bayesian model
Source: BMC Med Genomics. 2018 Oct 20;11:91. doi: 10.1186/s12920-018-0404-4 (PMC6195989; doi:10.1186/s12920-018-0404-4)
Supplement: Supplementary file 3 — Table S2. Effects of baseline selection on exon-level sensitivity and specificity. (PDF 46 kb) [file 12920_2018_404_MOESM3_ESM.pdf]

Table S2: Effects of baseline selection on exon-level sensitivity and specificity

Selected Genes	Mean Coverage (DMD-relative)	Sensitivity	Specificity
GFM1	5.358	0.974	0.996
GFM1, ACADM, FH, FASTKD2	1.804	0.961	0.998
GFM1, ACADM, FH, FASTKD2 AGL, SLC35D1, ALG6	0.880	0.961	0.998

Varying the number of genes included in the aggregate baseline seemed to have minimal effects on the exon-level sensitivity and specificity for the 15 female test samples. This suggests that the model can perform reasonably well even if suboptimal baseline targets are selected.
