# Supplementary material for: Clinical analysis of germline copy number variation in DMD using a non-conjugate hierarchical Bayesian model
Source: BMC Med Genomics. 2018 Oct 20;11:91. doi: 10.1186/s12920-018-0404-4 (PMC6195989; doi:10.1186/s12920-018-0404-4)
Supplement: Supplementary file 4 — Table S3. Coriell samples used for validation and supplemental experiments. (PDF 53 kb) [file 12920_2018_404_MOESM4_ESM.pdf]

Table S3: Coriell samples used for validation and supplemental experiments
*Used as negative control for software comparison only
†Used only for supplemental experiments (Figure S7)

ID	DMD Mutation	Sex
NA05117	EX45DEL	Female
NA04099	EX49-52DEL	Female
NA05159	EX46-50DEL	Female
NA07692	EX1-18DEL	Female
NA23087	EX2-30DUP	Female
NA23094	EX35-43DEL	Female
NA23099	EX8-17DUP	Female
NA04315	EX44DEL	Female
NA05264	7893delC (No CNV)	Female
NA07538*	None	Female
NA20239*	None	Female
NA04100†	EX49-52DEL	Male
NA23086†	EX2-30DUP	Male
