# Supplementary material for: Clinical analysis of germline copy number variation in DMD using a non-conjugate hierarchical Bayesian model
Source: BMC Med Genomics. 2018 Oct 20;11:91. doi: 10.1186/s12920-018-0404-4 (PMC6195989; doi:10.1186/s12920-018-0404-4)
Supplement: Supplementary file 5 — Figure S2. Read pair coverage for training and test samples. (PDF 483 kb) [file 12920_2018_404_MOESM5_ESM.pdf]

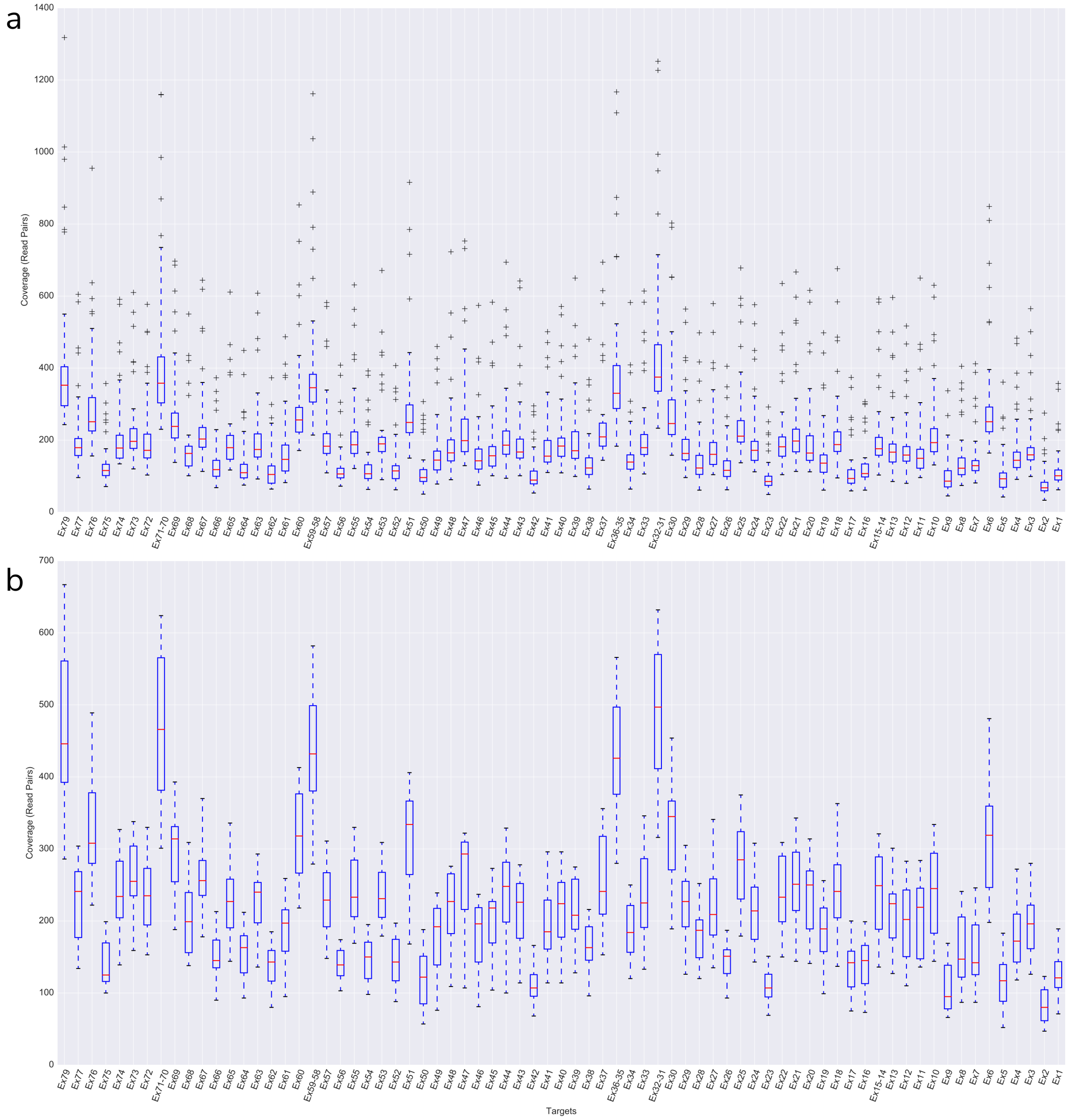

Figure S2: Read pair coverage for training and test samples Summary of coverage across primary transcript *DMD* exons for (a) 38 training samples and (b) 15 test samples (see Comparison to other software methods) . Boxes indicate first and third quartiles; red bands indicate medians. Whisker ends indicate data within 2.5 IQR of nearest quartile.
