# Supplementary material for: Clinical analysis of germline copy number variation in DMD using a non-conjugate hierarchical Bayesian model
Source: BMC Med Genomics. 2018 Oct 20;11:91. doi: 10.1186/s12920-018-0404-4 (PMC6195989; doi:10.1186/s12920-018-0404-4)
Supplement: Supplementary file 6 — Figure S3. Covariance estimation error. (PDF 202 kb) [file 12920_2018_404_MOESM6_ESM.pdf]

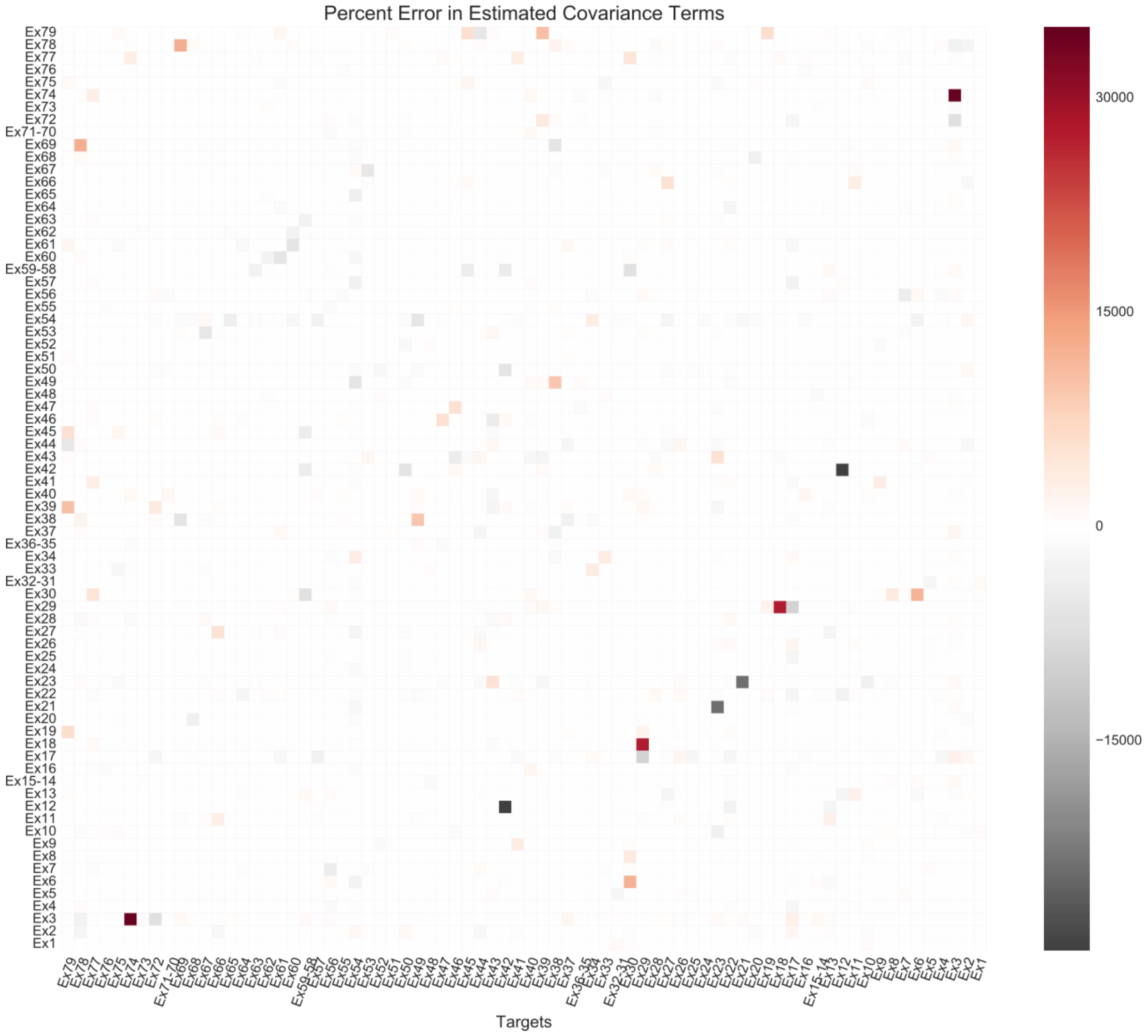

Figure S3: Covariance estimation error Plot of typical percent error across covariance matrix with $k = 79$, estimated from 35 simulated samples at coverage of 60,000 read pairs. Original covariance values derived from parameter estimates using training set of 38 samples as described in Methods. Target names represent primary transcript exons in *DMD* ordered by genomic position. Note that a small number of covariance terms have very high proportional error; the position of these terms is not consistent between different simulated instantiations of training cohorts.
