# Supplementary material for: Clinical analysis of germline copy number variation in DMD using a non-conjugate hierarchical Bayesian model
Source: BMC Med Genomics. 2018 Oct 20;11:91. doi: 10.1186/s12920-018-0404-4 (PMC6195989; doi:10.1186/s12920-018-0404-4)
Supplement: Supplementary file 7 — Figure S4. Covariance estimation error distributions. (PDF 169 kb) [file 12920_2018_404_MOESM7_ESM.pdf]

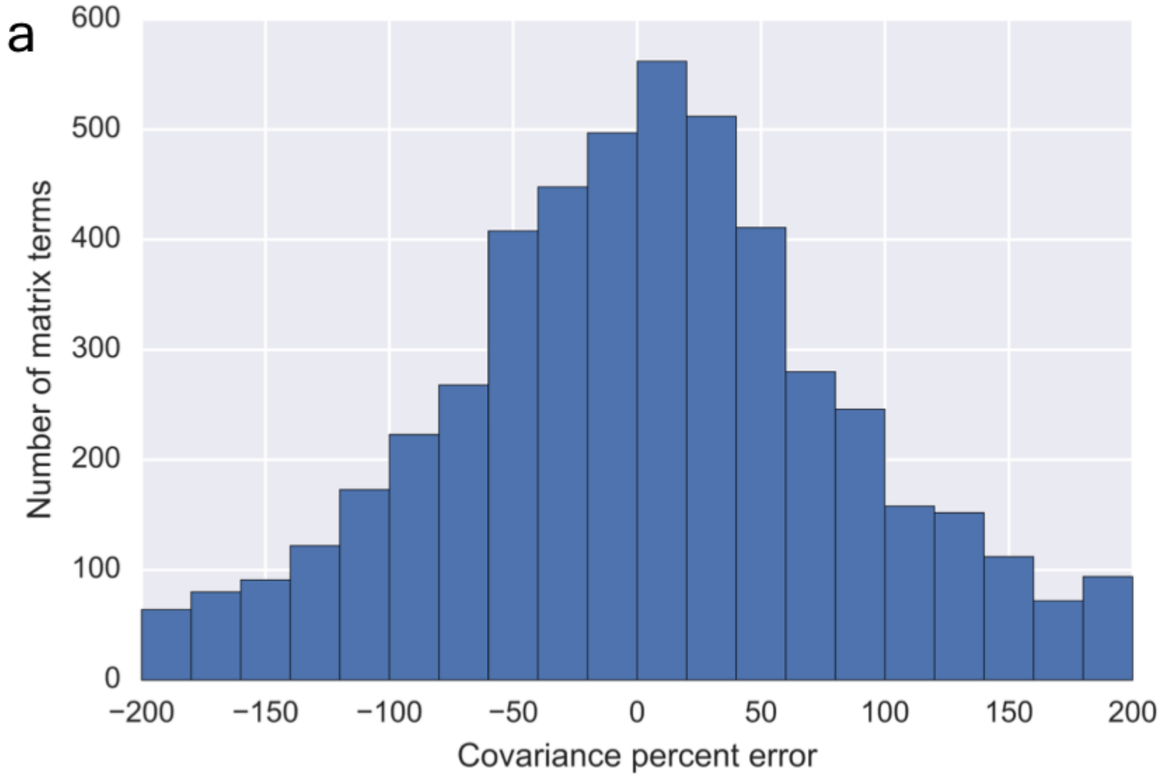

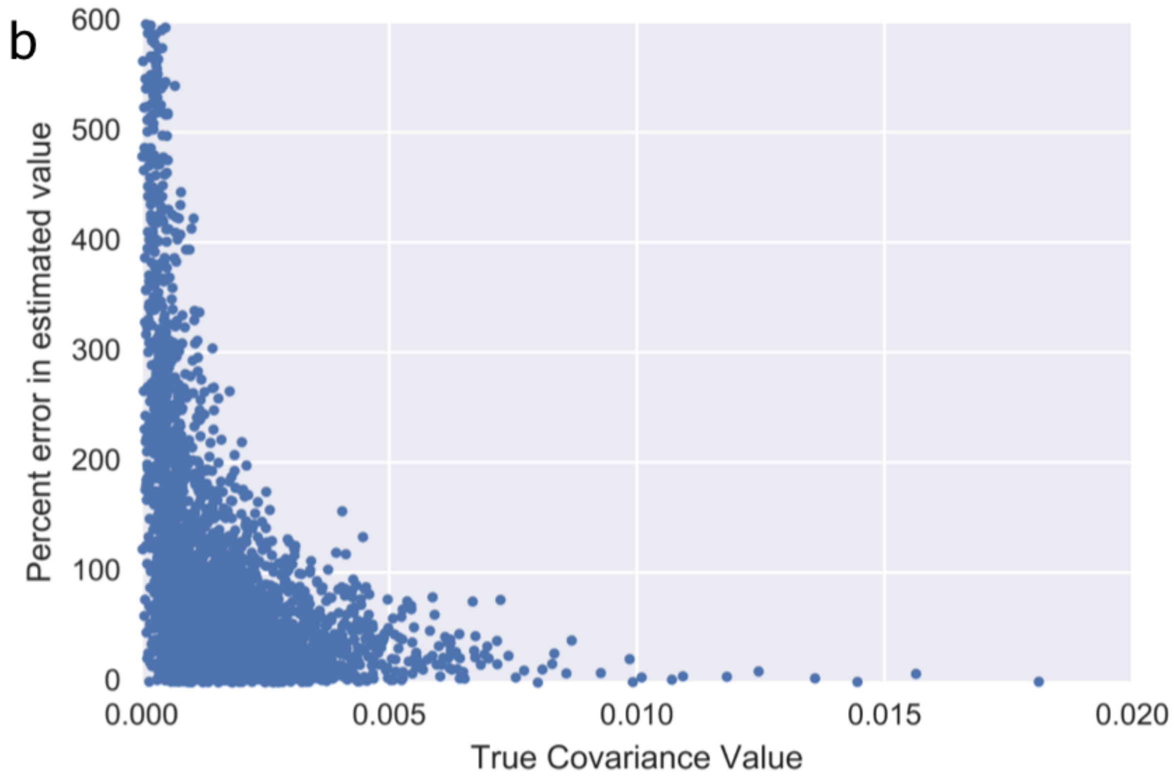

Figure S4: Covariance estimation error distributions (a) Distribution of covariance error proportions (excluding extreme outliers and distribution tail ends). 80% of all covariance terms are contained in this section of the distribution. (b) Plot showing inverse relationship between true covariance values and percent error in estimated values. Lower values are more likely to have higher proportional error.
