# Supplementary material for: Clinical analysis of germline copy number variation in DMD using a non-conjugate hierarchical Bayesian model
Source: BMC Med Genomics. 2018 Oct 20;11:91. doi: 10.1186/s12920-018-0404-4 (PMC6195989; doi:10.1186/s12920-018-0404-4)
Supplement: Supplementary file 8 — Figure S5. Results and coverage comparison for subject NA02387. (PDF 291 kb) [file 12920_2018_404_MOESM8_ESM.pdf]

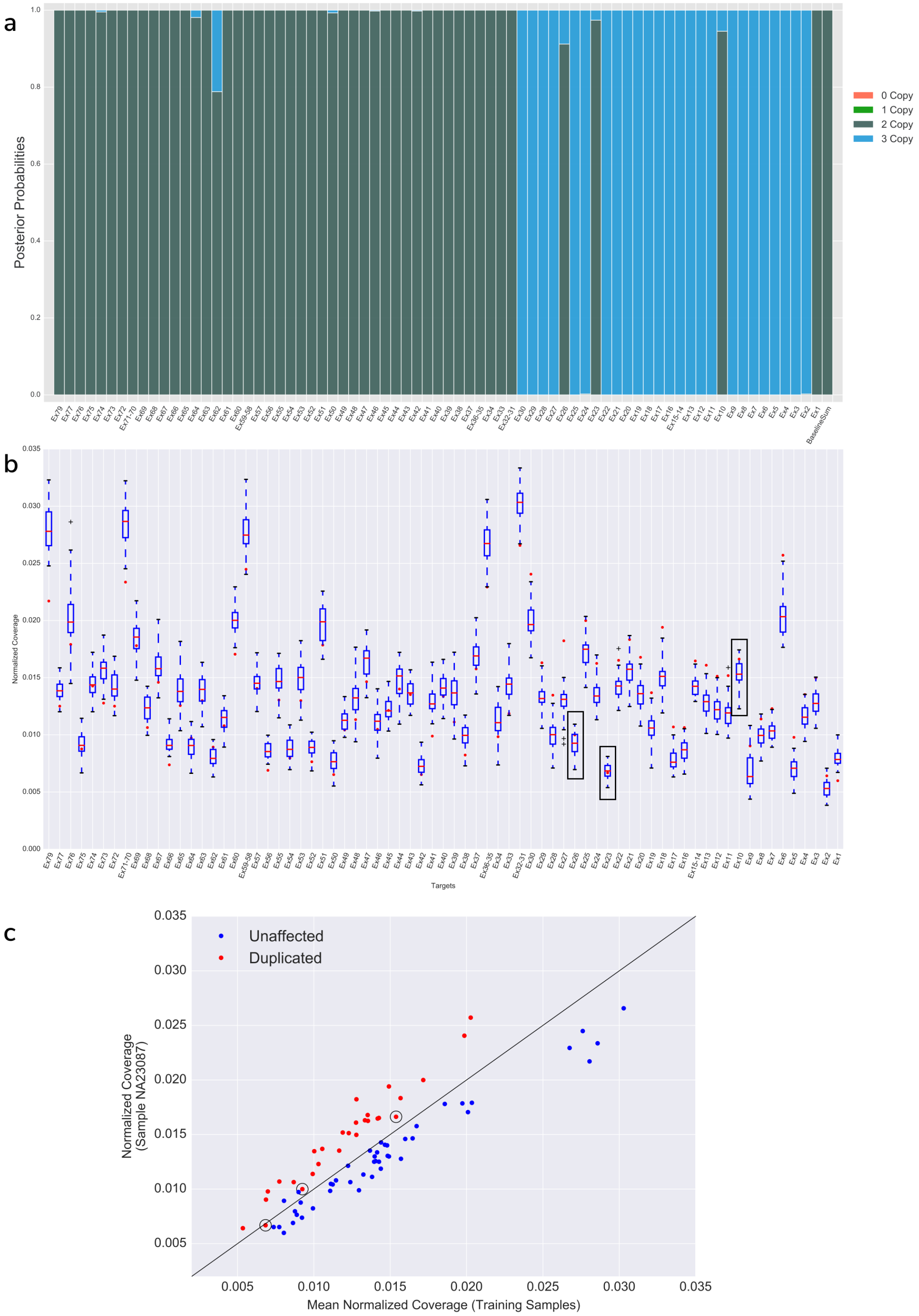

Figure S5: Results and coverage comparison for subject NA02387 Subject is known to carry duplication in Exons 2-30, largely detected by geneCNV, except for three exons with coverage levels very similar to those expected for normal copy number states (a) GeneCNV results including three non-contiguous exons (Exons 10, 23, 26) which were called as "normal" instead of duplicated (b) Normalized coverage comparison between distribution of training samples (boxes) and sample NA02387 (red dots). Normalized coverage is computed as proportion of individual target coverage relative to total coverage across *DMD*. Boxplot configured as in Figure S2. (c) Plot illustrating relationship between mean normalized training sample coverage and coverage for NA02387. Identity line displayed for reference and duplicated exons highlighted in red. Exons 10, 23, 26 marked for both (b) and (c).
