# Supplementary material for: Clinical analysis of germline copy number variation in DMD using a non-conjugate hierarchical Bayesian model
Source: BMC Med Genomics. 2018 Oct 20;11:91. doi: 10.1186/s12920-018-0404-4 (PMC6195989; doi:10.1186/s12920-018-0404-4)
Supplement: Supplementary file 9 — Figure S6. Estimation error with target number. (PDF 204 kb) [file 12920_2018_404_MOESM9_ESM.pdf]

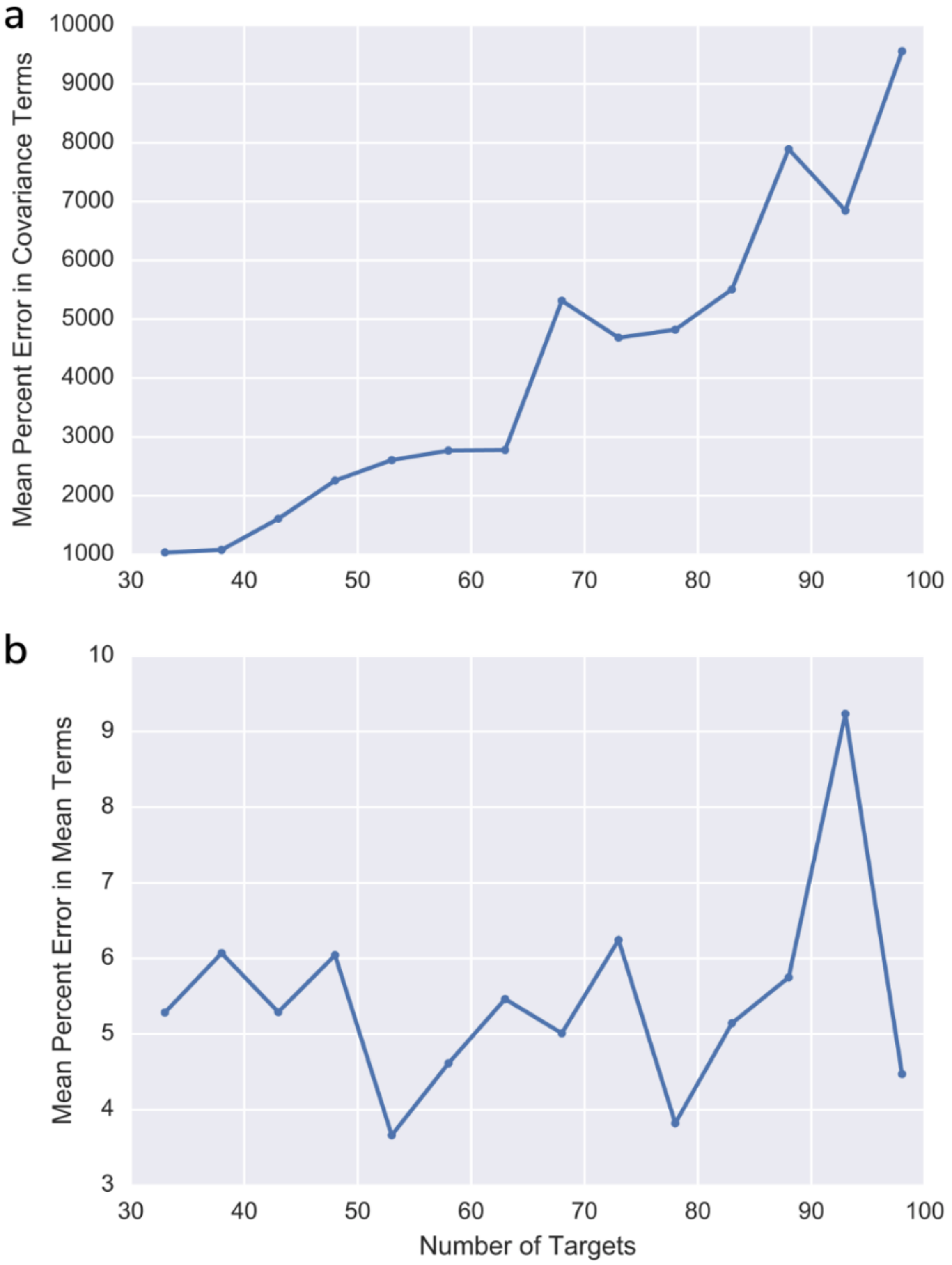

Figure S6: Estimation error with target number Plot showing average percent error in Σ (a) and μ (b) as the number of dimensions (targets) increases. At each target number k, mean vector and covariance matrix of the appropriate size $(k-1)$ and $(k-1) \times (k-1)$ were generated. One hundred samples with 500 reads/target were simulated using the true parameters, and used to recover the original values. Average error in covariance increases as the number of targets increases, though average error in mean does not correlate with number of targets.
