# Supplementary material for: Clinical analysis of germline copy number variation in DMD using a non-conjugate hierarchical Bayesian model
Source: BMC Med Genomics. 2018 Oct 20;11:91. doi: 10.1186/s12920-018-0404-4 (PMC6195989; doi:10.1186/s12920-018-0404-4)
Supplement: Supplementary file 10 — Figure S7. CNV identification in male subjects. (PDF 253 kb) [file 12920_2018_404_MOESM10_ESM.pdf]

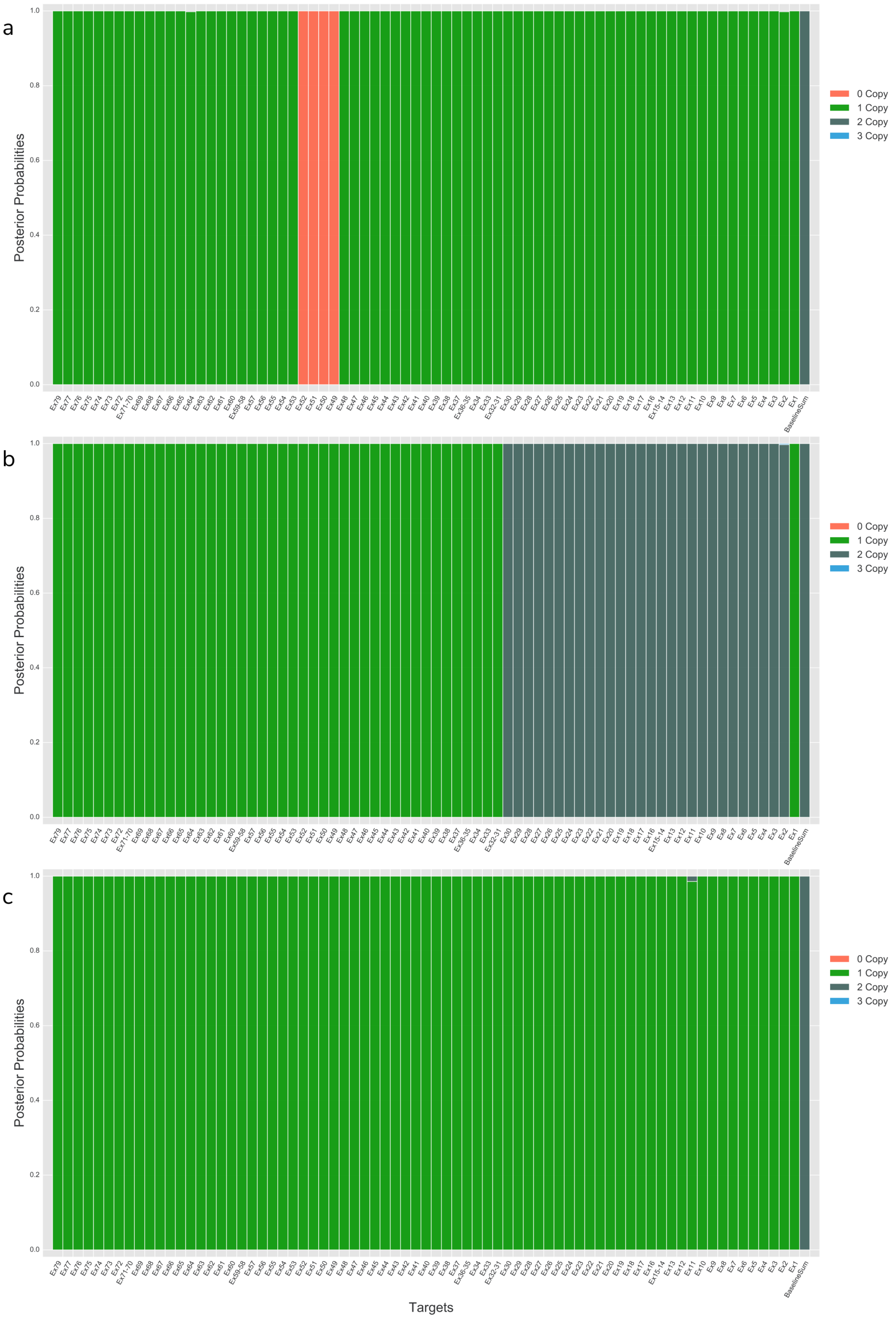

Figure S7: CNV identification in male subjects Results for male research and volunteer subjects using geneCNV trained on 38 female subject samples (a) Coriell subject NA04100 (EX49-52 deletion). (b) Coriell subject NA23086 (EX2-30DUP). (c) Volunteer subject with no known CNVs.
